# Supporting the Delivery of Total Knee Replacements Care for Both Patients and Their Clinicians With a Mobile App and Web-Based Tool: Randomized Controlled Trial Protocol

**DOI:** 10.2196/resprot.6498

**Published:** 2017-03-01

**Authors:** M Sazzad Hussain, Jane Li, Emily Brindal, Yasmin van Kasteren, Marlien Varnfield, Andrew Reeson, Shlomo Berkovsky, Jill Freyne

**Affiliations:** ^1^ Health and Biosecurity Commonwealth Scientific and Industrial Research Organization Epping, NSW Australia; ^2^ Health and Biosecurity Commonwealth Scientific and Industrial Research Organization Adelaide, SA Australia; ^3^ Flinders Digital Health Research Centre Flinders University Adelaide, SA Australia; ^4^ Australian e-Health Research Centre Commonwealth Scientific and Industrial Research Organization Herston, QLD Australia; ^5^ Data61 Commonwealth Scientific and Industrial Research Organization Acton, ACT Australia; ^6^ Data61 Commonwealth Scientific and Industrial Research Organization Sydney, NSW Australia

**Keywords:** orthopedic, TKR, rehabilitation, physiotherapy, mHealth, eHealth

## Abstract

**Background:**

Total knee replacement (TKR) surgeries have increased in recent years. Exercise programs and other interventions following surgery can facilitate the recovery process. With limited clinician contact time, patients with TKR have a substantial burden of self-management and limited communication with their care team, thus often fail to implement an effective rehabilitation plan.

**Objective:**

We have developed a digital orthopedic rehabilitation platform that comprises a mobile phone app, wearable activity tracker, and clinical Web portal in order to engage patients with self-management tasks for surgical preparation and recovery, thus addressing the challenges of adherence to and completion of TKR rehabilitation. The study will determine the efficacy of the TKR platform in delivering information and assistance to patients in their preparation and recovery from TKR surgery and a Web portal for clinician care teams (ie, surgeons and physiotherapists) to remotely support and monitor patient progress.

**Methods:**

The study will evaluate the TKR platform through a randomized controlled trial conducted at multiple sites (N=5) in a number of states in Australia with 320 patients undergoing TKR surgery; the trial will run for 13 months for each patient. Participants will be randomized to either a control group or an intervention group, both receiving usual care as provided by their hospital. The intervention group will receive the app and wearable activity tracker. Participants will be assessed at 4 different time points: 4 weeks before surgery, immediately before surgery, 12 weeks after surgery, and 52 weeks after surgery. The primary outcome measure is the Oxford Knee Score. Secondary outcome measures include quality of life (Short-Form Health Survey); depression, anxiety, and stress (Depression, Anxiety, and Stress Scales); self-motivation; self-determination; self-efficacy; and the level of satisfaction with the knee surgery and care delivery. The study will also collect quantitative usage data related to all components (app, activity tracker, and Web portal) of the TKR platform and qualitative data on the perceptions of the platform as a tool for patients, carers, and clinicians. Finally, an economic evaluation of the impact of the platform will be conducted.

**Results:**

Development of the TKR platform has been completed and deployed for trial. The research protocol is approved by 2 human research ethics committees in Australia. A total of 5 hospitals in Australia (2 in New South Wales, 2 in Queensland, and 1 in South Australia) are expected to participate in the trial.

**Conclusions:**

The TKR platform is designed to provide flexibility in care delivery and increased engagement with rehabilitation services. This trial will investigate the clinical and behavioral efficacy of the app and impact of the TKR platform in terms of service satisfaction, acceptance, and economic benefits of the provision of digital services.

**Trial Registration:**

Australian New Zealand Clinical Trials Registry (ANZCTR) ACTRN12616000504415; https://www.anzctr.org.au/Trial/Registration/TrialReview.aspx?id=370536 (Archived by WebCite at http://www.webcitation.org/6oKES0Gp1)

## Introduction

### Overview

Rates of total knee replacement (TKR) procedures have been rising steadily worldwide in recent years [1]. There has been an 80% increase in TKR procedures in Australia since 2003 [1-3], with increasing incidence and prevalence likely to continue due to factors such as the aging population, an increase in obesity and joint injury, and expectations of a continued physically active lifestyle, as well as higher demand for surgery at a younger age [1,4,5]. The mean age of TKR patients in Australia is 69 years; however, the demand for the surgery in the younger population (less than 65 years) has been increasing and is expected to double in the next decade [3,6-8].

Research has shown that a number of interventions prior to TKR surgery can improve outcomes or patient’s satisfaction [9,10]. Physiotherapy undertaken before TKR is effective in improving postoperative outcomes [10]. Quadriceps muscle stretching and upper body exercises before the procedure allow patients to be prepared for the postoperative condition and rehabilitation physiotherapy program [10]. Managing patient expectation prior to surgery has been shown to benefit the rehabilitation process and is an important predictor of postoperative outcomes [11,12]. To prepare patients for their TKR surgery and hospital stay, preoperative education may offer advantages for some patients when stratified according to their physical, psychological, and social conditions [13]. Moreover, interventions that help reduce comorbidities and obesity prior to surgery can have postoperative impact, such as improved recovery and reduced chances of adverse events [2].

Most TKR patients experience pain, joint stiffness, insufficient muscle strength, and limited physical activity after surgery [14]. Early mobility following surgery has been shown to improve functional mobility and prevent deep vein thrombosis [2,15]. Studies have shown that postoperative rehabilitation can improve short-term outcomes (3-4 months) with no significant difference between different types of treatment, however the benefits of longer term rehabilitation (4-12 months) are limited [16,17]. Postoperative physiotherapy (6-8 weeks) is common practice in Australia [1], but adherence to physiotherapy programs can be low. Adherence can be low because of pain during exercise and low levels of activity prior to surgery but also because of social and psychological issues such as low self-efficacy, depression, anxiety, and poor social support [18].

Clinicians require information from their patients for diagnosis and monitoring recovery. Many of these indicators are self-reported by the patients, such as completion of physiotherapy, functionality, pain, and sleep quality. Another key measure of progress used by physiotherapists and surgeons is range of motion (ROM), a measure of the flexibility of the knee joint [16].

We are looking at ways to motivate and assist patients to complete rehabilitation programs to realize proven benefits and improve TKR surgery outcomes. We propose a solution in the form of a digital orthopedic rehabilitation platform aimed at supporting patients and their clinicians across phases of TKR, from expectation management when considering TKR surgery right through to patient recovery and rehabilitation.

Our TKR platform comprises a mobile phone app used daily in conjunction with a wrist-worn activity tracker and a clinical Web portal. The aim is to address the challenges of engaging patients with information and physical exercise through self-managed tasks delivered via the app in surgical preparation and recovery and to bridge the communication gap between clinicians and patients via the clinical Web portal. The app is designed to assist patients in achieving meaningful behavior change around uptake, adherence, and completion of rehabilitation programs, along with meaningful education, self-monitoring, and behavior coaching through rich media content.

This paper introduces the TKR platform and details the research protocol of a study to determine the efficacy and associated impact of the platform in assisting patients in their preparation for and recovery from the TKR surgery. An Australian multisite (N=5) randomized controlled trial (RCT) will be conducted for this purpose. The duration of the study is expected to be 24 months, which includes in-hospital setup and staff training, participant recruitment, intervention delivery, and data analysis. The trial will run for 13 months for each patient.

### Total Knee Replacement Platform Technology

The design and functionality of the TKR platform have been informed through a user needs analysis. The user needs analysis (paper forthcoming) included interviews and focus groups with patients (n=11), general practitioners (n=8), orthopedic surgeons (n=12), and physiotherapists (n=3). The results of the analysis from the qualitative data clearly identified different stages of the TKR journey, each with different needs and priorities, which has informed the design of our platform technology for TKR. The qualitative data was used to refine features and functionalities of the platform, which comprises a patient facing app and activity tracker and a clinical Web portal for use by clinical care teams.

The information delivered via the app been scientifically validated and all exercise videos were created in conjunction with expert physiotherapists working with TKR patients.

In order to foster motivation, the purpose-built app for iOS and Android devices includes weekly psychoeducation sessions and tasks that are delivered by a program guide via text and voice recordings. This content is also reinforced through tasks (eg, goal-setting) that the user can complete outside of the app (eg, on paper). This motivational content was developed using self-determination theory [19] and included techniques designed to foster positive emotions [20]. Tools to assist in behavior change in the app use commonly identified behavior change techniques, such as self-monitoring, goal setting and reviewing, and rewarding/recognizing achievements that appear in various behavioral models which can be broadly encompassed by social cognitive theory [21,22].

**Figure 1:**
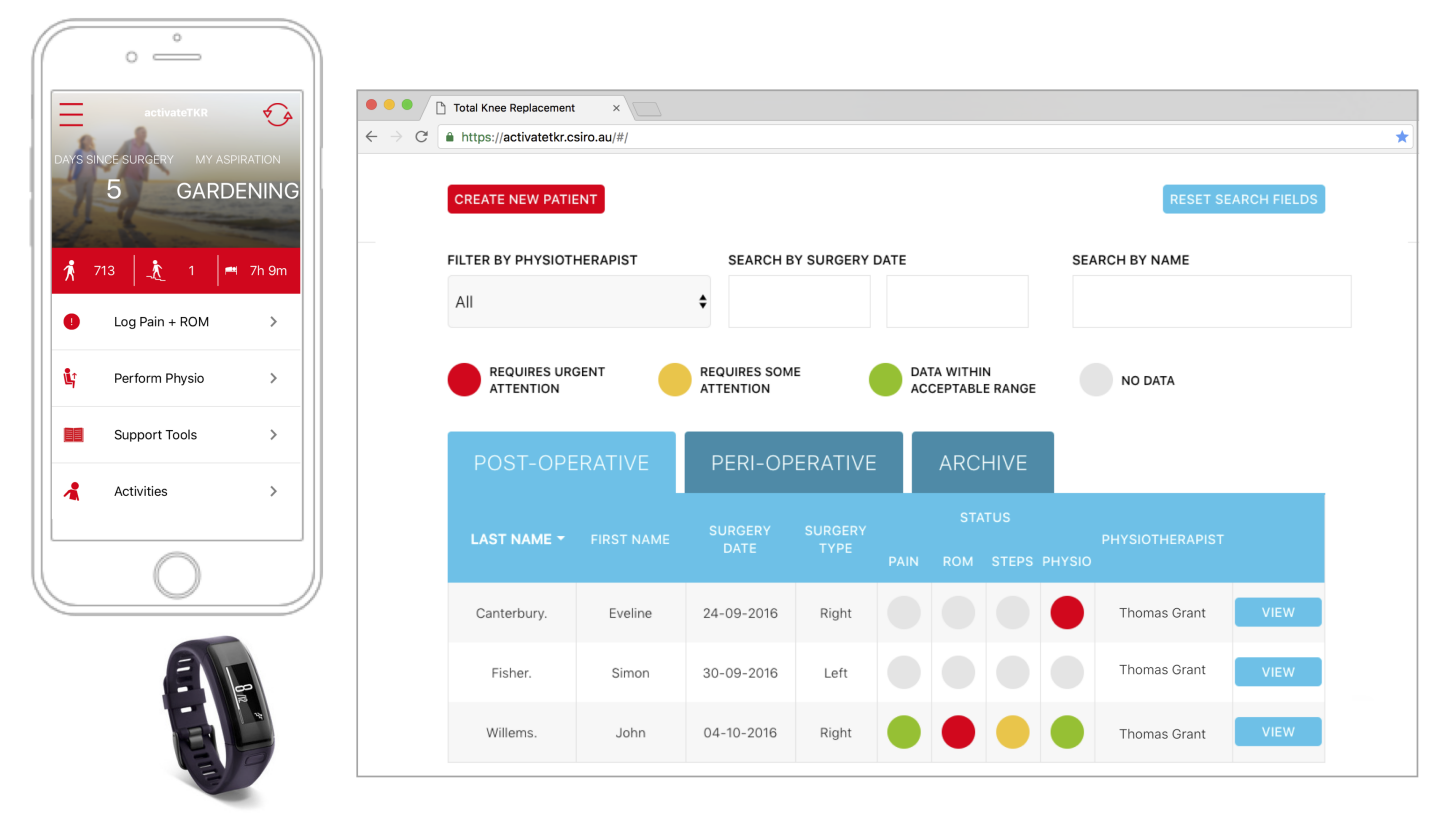
Total knee replacement platform. Left: app used daily in conjunction with a wrist-worn activity tracker. Right: clinical Web portal.

The clinical Web portal is designed for clinicians to monitor patient progress and configure physiotherapy programs. Patient data, gathered by the app, is transmitted to the portal for review by members of a patient care team. Physiotherapy programs for each patient are configured from a library of videos typically used for TKR rehabilitation. Once set, programs are available to patients in their app. Figure 1 provides sample screenshots of the TKR platform.

## Methods

### Overview and Aims

Data will be collected as part of a 13-month (for each participant), multisite unblinded RCT where participants are assigned to 1 of 2 study groups (1:1 allocation ratio). Both groups will undergo TKR surgery and be offered usual care and guidance from their surgeon and nominated health care team. In addition, the intervention group will receive the TKR app and a Garmin Vivosmart HR activity tracker. Patient participants will be scheduled for TKR surgery and managed through a project officer (PO), typically a member of the clinical team. Participants in the intervention group will be provided with instruction from the site PO in regard to using the technology. The aims of the study are as follows:

- To determine the efficacy of the TKR platform in delivering clinical and behavioral outcomes by specifically evaluating group differences
- To understand the impact of the TKR platform components and level of satisfaction with surgery and the care delivery
- To evaluate economic benefits

**Figure 2:**
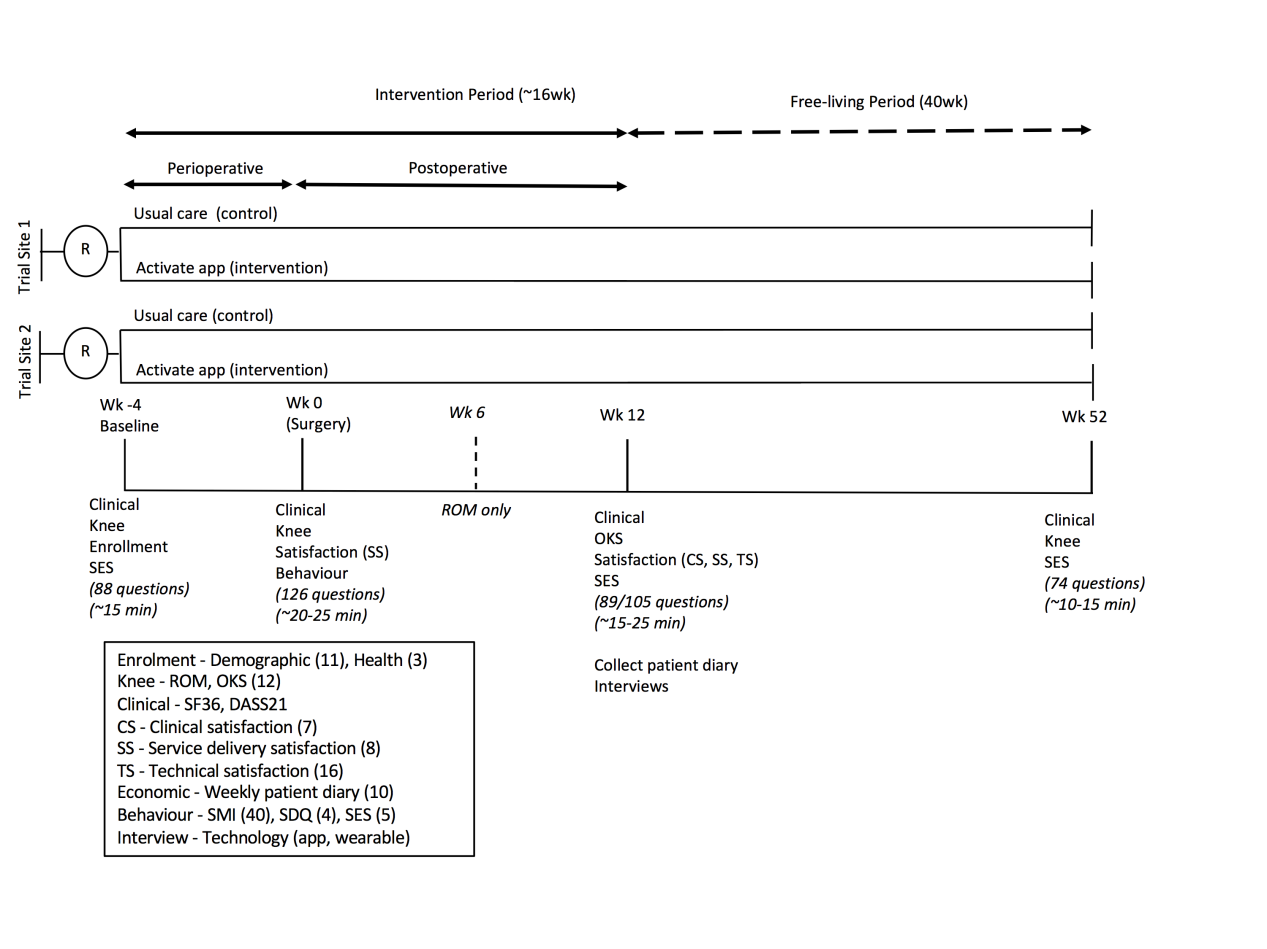
Study design: outcome measures, procedure, and timeline.

The study includes an active intervention period commencing when the patient is scheduled for surgery (approximately 4 weeks before surgery) and ending when the patient is 12 weeks postoperative. This is followed by a 40-week free-living period, finishing 1 year after surgery. Paper-based questionnaires will be completed by participants and the PO will be responsible for collecting responses from participants at 4 time points as illustrated in Figure 2.

All access to data will be controlled by authentication and authorization protocols designed to ensure the data is protected and only accessible by authorized persons. All information identifying the participants will be de-identified by a member of research team.

### Participants

#### Recruitment

**Figure 3:**
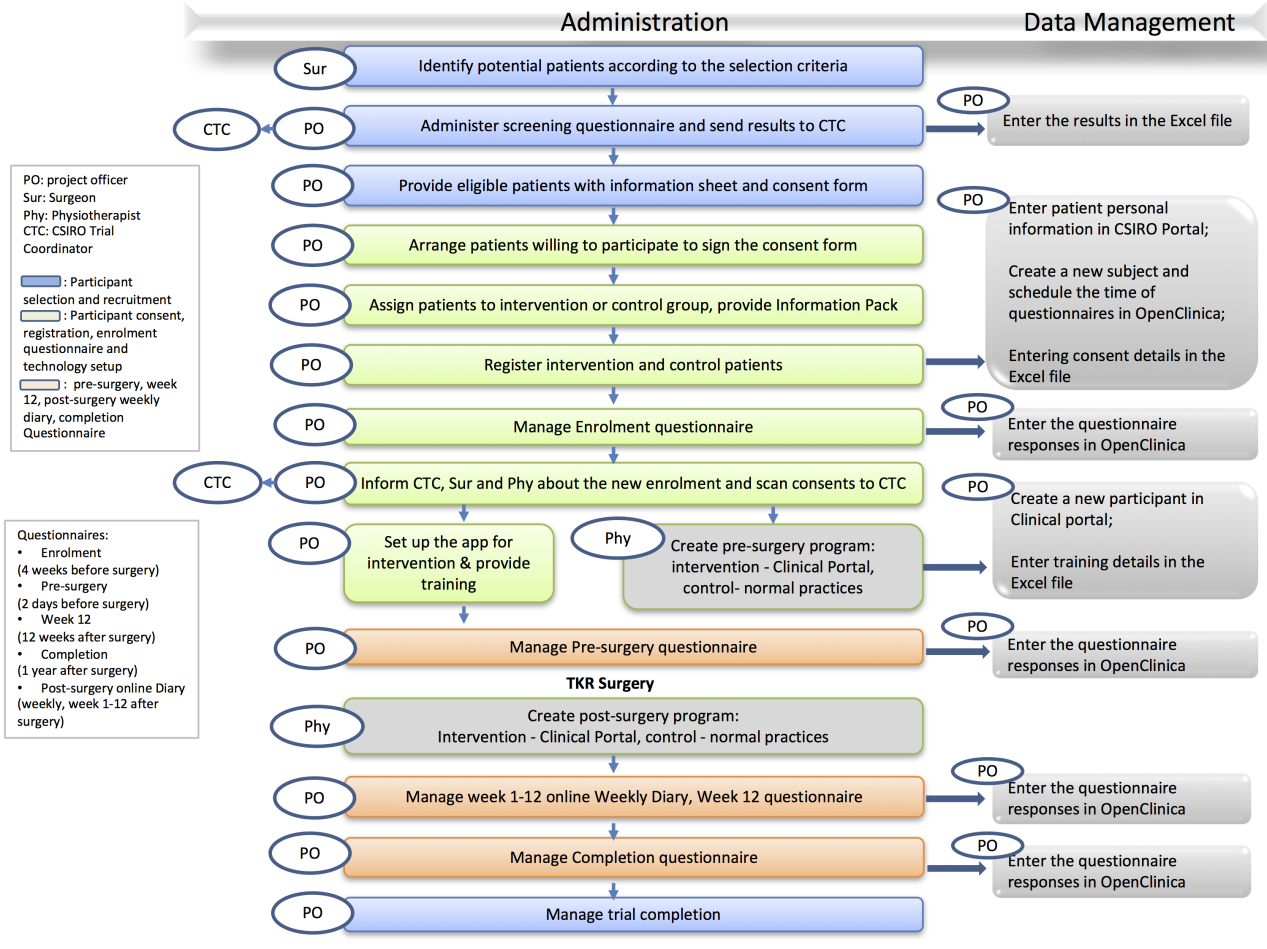
Process and workflow: patient recruitment, enrollment, trial data collection, and data administration.

Patients scheduled for TKR surgery at trial sites will be screened by surgeons for clinical eligibility. Those who meet eligibility requirements will be informed of the study by the site PO and screened for technical eligibility (eg, access to iPhone/iPad, smartphone/tablet, Internet access). Recruitment will be conducted by the PO and coordinated by the Clinical Trial Coordinator (CTC). Figure 3 summarizes the processes involved in the recruitment and enrollment of patients, trial data collection, and data administration.

#### Selection Criteria

Patients meeting the criteria as evaluated by the operating surgeon will be approached for recruitment by the PO. Inclusion criteria include:

- patients aged between 50 and 80 years
- suffering osteoarthritis as the principle diagnosis and reason for TKR
- scheduled to undergoing unilateral knee replacement (using commercial partner prosthesis) for the first time on one of the knees
- sufficiently healthy to not be adversely affected by participation in the trial
- deemed suitable candidates for physiotherapy both pre- and postsurgery
- have access to a smartphone/tablet (iOS or a leading Android-based device) with Internet connection via mobile Internet data or Wi-Fi connection
- willing to participate in the study and to have assessments on 4 occasions in line with scheduled appointments with their clinicians
- be sufficiently proficient in the English language to not be affected by participation in the trial

Exclusion criteria include:

- bilateral knee replacement
- previous unicompartmental replacement or tibial osteotomy on the same knee or previous lower extremity joint replacement surgery within the last 6 months
- major neurologic conditions or cognitive impairment that may result in inability to interact with the smartphone app
- uncontrolled diabetes, heart disease, or other medical conditions indicating participation in TKR is nonstandard

Based on the age criteria, surgeons will determine health-related eligibility of patients as well as assessing patient’s neurological condition using standard clinical practice.

### Randomization

#### Procedure

Eligible participants at each site will be randomized to 1 of the 2 treatment groups by a member of the research team. A simple randomization allocation technique in blocks of 10 using a randomization table generated by computer software will be used. Each site will have an intervention (ie, TKR platform) and a control group.

Participants will be randomized according to individual trial sites because usual care at each hospital (ie, trial site) will differ. Some hospitals offer presurgery education, others use online resources, and some offer daily outpatient rehabilitation classes or facilities such as hydrotherapy pools and gymnasiums. Other hospitals discharge patients to the community where they can privately engage services.

#### Intervention: Total Knee Replacement Platform

In stage 1, perioperative (typically 4 weeks before surgery) participants, upon receiving surgery date, will be provided with the TKR mobile app and a Garmin Vivosmart HR activity tracker at no cost. In stage 2, recovery (weeks 1-12 after surgery) participants will continue to use the TKR app and the activity tracker. Participants are expected to use the app daily and wear the activity tracker continuously (including while sleeping) for 16 weeks. Participants will continue to receive any prehabilitation and rehabilitation management as recommended by their surgeon, physiotherapist, or as provided by their hospital.

#### Control: Usual Care

Participants in the control group will receive the usual inpatient and outpatient prehabilitation and rehabilitation services as recommended by their surgeon, physiotherapist, or as provided by their hospital.

### Outcome Measures

#### Overview

Basic demographic data and a brief history of medical/health conditions will be taken at baseline. PO will be responsible for collecting responses from participants, either face-to-face at the clinic or received by post. OpenClinica (www.openclinica.com) will be used to store digital copies of responses from self-reported questionnaires, which include validated and trial specific questionnaires. The patient diaries and audit questions for health care providers will inform the economic outcomes for this study. Patient diaries will be documented online using SurveyGizmo (www.surveygizmo.com).

Interviews will be conducted with a selected number of patients, their carers, and clinicians in order to capture outcomes related to the TKR platform technology with regard to service delivery satisfaction and usability. Table 1 summarizes the outcome measures, the data collection tools and procedures, and the time points. The remainder of this section details the various outcome measures.

**Table 1:** Outcome measures, data collection tools and procedures, and timeline.

Outcome		Measurement tool/procedure/ data collection	Time points (weeks)			
			*Baseline*	*0*	*12*	*52*
**Patient self-report**						
	Knee	OKS^a^	x	x	x	x
	Quality of life	SF-36^b^	x	x	x	x
	Depression, anxiety, stress	DASS21^c^	x	x	x	x
	Clinical satisfaction	Trial specific questionnaire			x	
	Service delivery satisfaction	Trial specific questionnaire		x	x	
	Technical satisfaction	Trial specific questionnaire			x	
	Self-motivation	SMI^d^		x		
	Self-determination	SDQ^e^		x		
	Self-efficacy	SES^f^	x	x	x	x
	Economic	Patient diary			x	
**By clinicians or project officer**						
	ROM^g^	Goniometer	x	x	x^h^	x
**Structured interviews**						
	Service satisfaction (patients, carers, clinicians)				x	
	Technical/usability (patients, carers, clinicians)				x	
**Audit**						
	Health care provider audit	Trial specific questionnaire			x	

^a^OKS: Oxford Knee Score.

^b^SF-36: Short-Form Health Survey.

^c^DASS21: Depression, Anxiety, and Stress Scale.

^d^SMI: Self-Motivation Inventory.

^e^SDQ: Self-Determination Questionnaire.

^f^SES: Self-Efficacy Scale.

^g^ROM: range of motion.

^h^At 6 weeks.

#### Primary Outcome

The primary outcome measure is self-reported knee pain and function as measured by the Oxford Knee Score (OKS) [23]. The OKS is 12-item questionnaire with 5 items for assessing pain and 7 for assessing function, each with 5 categories of response. Each item is scored from 1 to 5, from least to most difficulty or severity. A single combined score ranges from 12 (ie, least difficult) to 60 (ie, most difficult), and a lower score indicates a better outcome. The OKS has been developed and validated to specifically assess function and pain after TKR and is widely used by clinicians.

#### Secondary Outcomes

The secondary outcomes include:

- Quality of life (RAND 36 Item Short-Form Health Survey [SF-36]): SF-36 is a 36-item questionnaire that will be used for constructing summary scores of physical and mental components [24]. The questionnaire assesses 8 domains of wellness through subscales including body pain, physical function, general health, mental health, social functioning, and emotional role. The SF-36 is a popular instrument in the knee domain to measure generic quality of life [25].
- Knee range of motion (ROM): A long-arm goniometer will be used for measuring the knee ROM (flexion and extension) [26]. The goniometer axis will be positioned over the lateral knee joint area and the arms will be aligned with the lateral malleolus using the greater trochanter. The active knee flexion will be measured in the sitting position and the active knee extension will be measured in the long sitting or supine position. ROM as part of week 12 will be collected during the patients’ follow-up clinical visit, typically 6 weeks following surgery.
- The Depression, Anxiety and Stress Scale (DASS21) will be used to measure mental well-being. This is a 21-item questionnaire using a 4-point Likert scale with 7 items per condition (depression, anxiety, stress) [27]. Each item has equal weighting (0 to 3) and a higher score indicates greater symptoms. While SF-36 covers general mental health (psychological distress and psychological well-being), DASS21 is specifically designed to measure negative emotional states and severity of feelings related to depression, anxiety, and stress and has proven to be sensitive to patients’ response to treatment.
- Satisfaction with knee surgery and service delivery (prehabilitation, rehabilitation) and the use of technology (eg, app and wearable) will be measured with questionnaires developed by the Commonwealth Scientific and Industrial Research Organization (CSIRO) using a 5-point Likert scale. The questionnaires are designed to capture surgical results, surgical recommendations, interaction with clinicians, empowerment experience, observability, and overall satisfaction with the service delivery. The technical satisfaction questionnaire will capture complexity, compatibility factors, and experience with the app and the wearable.
- Self-motivation will be captured using a validated 40-item scale [28] called the Self-Motivation Inventory (SMI). It has been used previously to assess rehabilitation outcomes for anterior cruciate ligament reconstruction [29]. The scale captures 10 areas of self-motivation and could be a critical predictor of adherence to a rehabilitation program. Therefore, we will be capturing this to control for any confounding effects that this may have on the experimental conditions.
- Self-determination will be captured using a 4-item questionnaire in order to assess whether the intervention group has higher levels of autonomous and/or intrinsic motivation after the presurgery phase (ie, week 0). In line with methods of Sheldon and Kasser [30], participants will be asked to rate 4 reasons for pursuing the goal of successful rehabilitation according to how much each describes their reason for having this goal. These reasons cover the proposed spectrum of possible motivations from external to introjected to autonomous to intrinsic, all derived from self-determination theory. All responses are rated from 1 (not at all because of this reason) to 9 (completely because of this reason).
- Self-efficacy to perform rehabilitation activities is a critical predictor of rehabilitation success. It describes a person’s confidence in their ability to perform certain behavior in the face of challenges. In order to capture changes in self-efficacy, we will be using an adapted version of a physical activity Self-Efficacy Scale (SES) [31] designed to ask these questions around rehabilitation physiotherapy exercises rather than general exercise intention. This is a 5-item questionnaire using a 4-point Likert scale.

#### Technical and Usage Outcomes

Usage logs for the TKR platform (app, activity tracker, and Web portal) will be gathered and analyzed to help understand technology uptake and feature usage by patients and clinicians. Correlations and dependencies between usage and outcomes will be evaluated. Self-reported compliance with physiotherapy programs, content access, activity tracker data, and goal achievements collected from the app will also be analyzed.

#### Health Economic Outcomes

The economic evaluation will assess the costs and benefits accruing to both patients and service providers. It will also consider broader economic impact such as impact on carers’ time, ability to work, and other health system costs (eg, visits to general practitioners).

For service providers, we will estimate the delivery costs of group or one-on-one classes that deliver physiotherapy intended for self-guided physiotherapy including staff time, space, and equipment costs. Each trial site will complete a health care provider audit form for this purpose. Further to this, we will note the time taken to review patient data through the TKR clinical Web portal, time taken on patient phone calls, etc.

For patients and their carers, we focus on the direct and indirect costs, such as impact on work or spare time, various services patients receive from the community, visits they make to see their health care professional, visits related to any nonclinical services, and an estimate of out-of-pocket expense. In most cases patients will not be asked to estimate monetary costs as these are highly variable and often subsidized (eg, by government or health insurers). The research team will assign generic costs to each activity or impact for the analysis. This approach allows the costs and benefits to other stakeholders such as government and health insurers to also be compared across the 2 treatment groups.

#### Interview Participants

Using a maximum variation sampling [32] technique, intervention group patients (n=20), carers (n=10), and surgeons/physiotherapists (n=10) will be recruited by the site POs with guidance from the CTC for interview. Previous experience suggests that theoretical saturation will be reached with these numbers and a pool of diverse participants will be identified. Patient diversity will focus on gender, age (over 18 years only), TKR experience, education level, and private versus public health care. Carer sampling will target spouses, children, and friends of patients, with a balance of gender and age. All participating clinicians will be invited for interview by a researcher.

Interviews will be semistructured. Interviews will be audiorecorded and transcribed and analyzed using NVivo (QSR International) software. Interviews with patients and carers will focus on technology acceptance, their experience with the app, and what features they liked and disliked. Interviews with surgeons and physiotherapists will focus on features of the clinical Web portal and the usefulness of the portal for monitoring patients postoperatively.

Inclusion criteria for carers include the following: be the person who is nominated as the primary carer after hospital discharge of a patient participating in the TKR study from the intervention group (ie, using the app and activity tracker), be sufficiently healthy to not be adversely affected by the participation in the interview or study, and have the willingness to participate in the study and be interviewed. Clinicians must have provided care to at least 1 patient participant in the TKR study to take part in the interview.

Carers and clinicians participating in an interview will be sent the information sheet and consent form by email or post, separate from the ones used for patients. Paper-based consent will be obtained in face-to-face interviews, and verbal consent will be obtained for telephone interviews.

Estimated times for patient and carer interviews are 30 to 45 minutes, and estimated time for clinicians is 45 to 60 minutes.

### Sample Size

The sample size is calculated based on the primary outcome (ie, OKS), by considering 80% power (*P*=.05) with a 15% difference in the control group and the intervention group. The standard deviation within each group in the TKR trials can vary based on factors such as number of trial sites (eg, single-center vs multicenter) and heath care cover (eg, public patients vs private patients). Furthermore, in a repeated measure scenario or when considering multiple assessments within a trial (eg, baseline, prehabilitation, 12 weeks rehabilitation, 12 months free living), the correlation among repeated measures needs to be considered as well. Based on these factors the sample size is presented in Table 2 for a variety of scenarios. A low subject variation can be expected with participants when considering only 1 trial site or 1 type of health care cover (eg, public), and a high variation can be expected when participants are mixed. A high correlation in repeated measures can be expected for patients with big differences in the outcome measure within the assessment points.

**Table 2:** Estimated sample size (with 20% loss) based on standard deviation (SD) within groups and correlation among baseline and 3 repeated measures [80% power, *P*=.05, 15% difference].

SD within group/ correlation repeated measure	Low repeated correlation (SD 0.2)	Mid repeated correlation (SD 0.5)	High repeated correlation (SD 0.8)
Low subject variation (SD 0.2)	31	46	60
Mid subject variation (SD 0.5)	170	262	360
High subject variation (SD 0.8)	432	674	914

We estimate that a total of 262 patient participants would be sufficient for this study; however, we expect a maximum of 320 participants from the 5 trial sites based on their rate of TKR surgeries during the trial timeline. A minimum sample size of 100 is suggested for using OKS as the primary outcome measure [33]. Based on the literature [1,7,34], 80% power (*P*=.05) with a 15% difference in the control group and the intervention group has been reported for primary outcome measure.

### Statistical Analysis

Statistical data analysis will be performed using an intention-to-treat approach. Mixed model analysis will be used for continuous variables (primary and secondary outcomes) measured at week 0, week 12, and week 52 for those receiving the TKR platform compared with usual care by time effects. Fixed effects will be included for the intervention, the time points, and the interaction between the 2 variables. These models will also account for relevant covariates such as baseline (4 weeks before surgery) measures, participant sex, baseline levels of motivation, etc. These covariates will be entered as fixed effects. Due to potential confounding effects between different hospitals, these will be also assessed in the mixed model as random effects. Sensitivity analysis will be used to assess the impact of these covariates before the final model is constructed. Other outcome measures of app usage, self-management (eg, physical activity), and compliance with the program (eg, physiotherapy exercise) will be analyzed using logistic or conditional logistic regression to adjust the comparisons for other variables for categorical or ordinal variables and multiple regression for continuous variables. All statistical analysis will be done using Excel (Microsoft Corp), R (R Foundation), or SPSS (IBM Corp).

### Timelines

**Figure 4:**
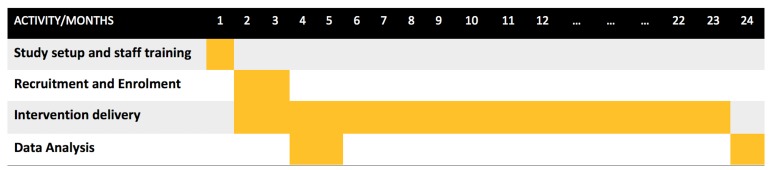
Research timelines.

It is intended that the total study will run for 24 months. This is subject to patient (ie, participants) recruitment. Data capture pertaining to each patient is required for 13 months. Figure 4 presents the expected timeline for this research.

## Results

This research is being conducted by CSIRO and cofunded by commercial partner Johnson & Johnson Medical Australia. The research protocol is approved by the CSIRO and Mater Health Services human research ethics committees.

The development of the TKR platform is complete, and the technology has been deployed for trial. The trial commenced at Gosford Private Hospital in October 2016, and all other sites are expected to come on board early in 2017 once training of relevant trial site staff (ie, POs, surgeons, physiotherapists, fellows) is complete.

## Discussion

Variations in the provision of care by health care providers, rising health care costs, and the increased uptake of TKR surgery have recently contributed to the need to ensure that TKR is effective, efficient, and safe as this can have a significant impact on patient satisfaction, medical costs, and access to health care service [35,36]. Rehabilitation programs after TKR surgery are designed to assist in recovery, restoring functional independence through physiotherapy programs and other interventions supporting the process. The programs have been popular in Western countries with both inpatient and outpatient settings.

It is evident that rehabilitation programs are costly and mostly accessed by private health care patients. Patients generally have the option of inpatient or outpatient rehabilitation after TKR. Inpatient rehabilitation clinics are only available in large metropolitan areas and because of the cost only an option for private insured patients [37]. Outpatient rehabilitation usually commences immediately after hospital discharge, which provides supervised (ie, one-on-one or group) exercises to the patients by a physiotherapist and is completed within 6 to 8 weeks after TKR surgery [7,38]. Outpatient rehabilitation has a large component of self-managed exercise at home. Compliance with in-home exercise is a recognized challenge in physiotherapy [39]. Compliance with rehabilitation physiotherapy after TKR is dependent on motivation or self-determination [18].

Our study will test whether a digital orthopedic rehabilitation platform comprising a smartphone-based program with a wearable activity tracking device and a clinical Web portal where clinicians monitor patients can assist TKR patients and their carers in the TKR journey. The proposed platform will provide flexibility, particularly in rural, remote, or busy lifestyles, and has the potential to achieve the same clinical outcomes as normal business as usual care.

Potential barriers to participant adaptation of the intervention may include a lack of technology/eHealth literacy for the targeted age group, contributing to the unwillingness to continue using the app and the wearable activity tracker. The trial is a research project rather than a new health service. It requires clinicians and POs at trial sites to help participants follow the protocol while integrating the intervention into their usual care model.

## Abbreviations

CSIRO: Commonwealth Scientific and Industrial Research Organization
CTC: clinical trial coordinator
DASS21: Depression, Anxiety, and Stress Scale
OKS: Oxford Knee Score
PO: project officer
RCT: randomized controlled trial
ROM: range of motion
SDQ: Self-Determination Questionnaire
SES: Self-Efficacy Scale
SF-36: Short-Form Health Survey
SMI: Self-Motivation Inventory
TKR: total knee replacement

## Appendix

Multimedia Appendix 1 Questionnaires (i.e. instruments) and interview questions.
