# Supplementary material for: Supporting the Delivery of Total Knee Replacements Care for Both Patients and Their Clinicians With a Mobile App and Web-Based Tool: Randomized Controlled Trial Protocol
Source: JMIR Res Protoc. 2017 Mar 1;6(3):e32. doi: 10.2196/resprot.6498 (PMC5352858; doi:10.2196/resprot.6498)
Supplement: Multimedia Appendix 1 [file resprot_v6i3e32_app1.pdf]

Appendix A **Evaluation Measures**

A.1 Patient/Participant Enrolment

A.1.1 Demographics

Total number of questions: 9

1. Date of Birth (dd/mm/yyyy): ________________________
2. Gender: (Please circle) Male Female Other
3. Height: _________________ cms
4. Weight: ____ kgs
5. Do you live alone or with others? (Please circle) Alone With others

   If answered 'Alone', have you nominated a primary carer after your TKR surgery?

   (Please circle) Yes No
6. Do you currently have private health insurance? (Please circle) Yes No
7. What is your highest level of education?
   - ✓ Less than Year 12 or equivalent
   - ✓ Year 12 or equivalent
   - ✓ Vocational qualification
   - ✓ Associate diploma
   - ✓ Undergraduate diploma
   - ✓ Bachelor degree (including honours)
   - ✓ Postgraduate diploma
   - ✓ Master's degree
   - ✓ Doctorate
8. Occupation: ________________________
9. What is your employment status?
   - ✓ Employee, full time
   - ✓ Employee, part time
   - ✓ Self-employed
   - ✓ Not in paid employment due to retirement
   - ✓ Not in paid employment for other reasons

A.1.2 Medical/Health Condition

Total number of questions: 3

1. When was your osteoarthritis diagnosed? .... mm/yyyy

2. Which of the following **medical conditions** has been diagnosed by a **doctor**? Please tick (✓) all appropriate boxes

☐High blood pressure ☐HIV ☐Epilepsy
☐Kidney or Liver disease ☐Angina ☐Asthma
☐Pernicious anaemia ☐Stroke ☐Gastrointestinal disorders/bowel conditions
☐High cholesterol ☐Thyroid ☐Cancer
☐Heart disease ☐Migraine ☐Known allergies
☐Type 1 diabetes ☐Gout ☐Chronic obstructive pulmonary disease
☐Type 2 diabetes ☐Hepatitis A/B/C
Other Medical Condition.. (textbox)

3. If you've had major surgery please give details and indicate year if known
.... (textbox)

A.2 Knee Specific Outcome Measures

A.2.1 Oxford Knee Score (OKS)

Total number of questions: 12

During the past 4 weeks......	*1*	*2*	*3*	*4*	*5*
1. How would you describe the pain you usually have from your knee?	None	Very mild	Mild	Moderate	Severe
2. Have you had any trouble with washing and drying yourself (all over) because of your knee?	No trouble at all	Very little trouble	Moderate trouble	Extreme difficulty	Impossible to do
3. Have you had any trouble getting in and out of a car or using public transport because of your knee? (whichever you tend to use)	No trouble at all	Very little trouble	Moderate trouble	Extreme difficulty	Impossible to do
4. For how long have you been able to walk before pain from your knee becomes severe? (with or without a stick)	No pain/More than 30 minutes	16 to 30 minutes	5 to 15 minutes	Around the house only	Not at all/pain severe when walking
5. After a meal (sat at a table), how painful has it been for you to stand up from a chair because of your knee?	Not at all painful	Slightly painful	Moderately pain	Very painful	Unbearable
6. Have you been limping when walking, because of your knee?	Rarely / never	Sometimes or just at first	Often, not just at first	Most of the time	All of the time
7. Could you kneel down and get up again afterwards?	Yes, easily	With little difficulty	With moderate difficulty	With extreme difficulty	No, impossible
8. Have you been troubled by pain from your knee in bed at night?	No nights	Only 1 or 2 nights	Some nights	Most nights	Every night

9. How much has pain from your knee interfered with your usual work (including housework)?	Not at all	A little bit	Moderately	Greatly	Totally
10. Have you felt that your knee might suddenly 'give way' or let you down?	Rarely / Never	Sometimes or just at first	Often, not at first	Most of the time	All of the time
11. Could you do the household shopping on your own?	Yes, easily	With little difficulty	With moderate difficulty	With extreme difficulty	No, impossible
12. Could you walk down one flight of stairs?	Yes, easily	With little difficulty	With moderate difficulty	With extreme difficulty	No, impossible

A.3 Clinical Outcome Measures

A.3.1 RAND Short Form Health Survey (SF-36) Questionnaire Items

Total number of questions: 36

	Excellent (1)	*Very good (2)*	*Good (3)*	*Fair (4)*	*Poor (5)*
1. In general, would you say your health is:					

	Much better now than one year ago (1)	*Somewhat better now than one year ago (2)*	*About the same (3)*	*Somewhat worse now than one year ago (4)*	*Much worse now than one year ago (5)*
2. Compared to one year ago, how would your rate your health in general now?					

The following items are about activities you might do during a typical day. Does your health now limit you in these activities? If so, how much?	*Yes, Limited a Lot (1)*	*Yes, Limited a Little (2)*	*No, Not limited at All (3)*
3. Vigorous activities, such as running, lifting heavy objects, participating in strenuous sports			
4. Moderate activities, such as moving a table, pushing a vacuum cleaner, bowling, or playing golf			
5. Lifting or carrying groceries			
6. Climbing several flights of stairs			
7. Climbing one flight of stairs			
8. Bending, kneeling, or stooping			
9. Walking more than a mile (1.6 km)			
10. Walking several blocks			
11. Walking one block			
12. Bathing or dressing yourself			

During the past 4 weeks, have you had any of the following problems with your work or other regular	*Yes (1)*	*No (2)*

daily activities as a result of your physical health?		
13. Cut down the amount of time you spent on work or other activities		
14. Accomplished less than you would like		
15. Were limited in the kind of work or other activities		
16. Had difficulty performing the work or other activities (for example, it took extra effort)		

During the past 4 weeks, have you had any of the following problems with your work or other regular daily activities as a result of any emotional problems (such as feeling depressed or anxious)?	*Yes (1)*	*No (2)*
17. Cut down the amount of time you spent on work or other activities		
18. Accomplished less than you would like		
19. Didn't do work or other activities as carefully as usual		

	Not at all (1)	*Slightly (2)*	*Moderately (3)*	*Quite a bit (4)*	*Extremely (5)*
20. During the past 4 weeks, to what extent has your physical health or emotional problems interfered with your normal social activities with family, friends, neighbours, or groups?					

	None (1)	*Very mild (2)*	*Mild (3)*	*Moderate (4)*	*Severe (5)*	*Very severe (6)*
21. How much bodily pain have you had during the past 4 weeks?						

	Not at all (1)	*Slightly (2)*	*Moderately (3)*	*Quite a bit (4)*	*Extremely (5)*
22. During the past 4 weeks, how much did pain interfere with your normal work (including both work outside the home and housework)?					

These questions are about how you feel and how things have been with you during the past 4 weeks. For each question, please give the one answer that comes closest to the way you have been feeling.	*All of the Time (1)*	*Most of the Time (2)*	*A Good Bit of the Time (3)*	*Some of the Time (4)*	*A Little of the Time (5)*	*None of the Time (6)*
23. Did you feel full of pep?						
24. Have you been a very nervous person?						
25. Have you felt so down in the dumps that nothing could cheer you up?						
26. Have you felt calm and peaceful?						
27. Did you have a lot of energy?						
28. Have you felt downhearted and blue?						

29. Did you feel worn out?						
30. Have you been a happy person?						
31. Did you feel tired?						

	All of the time (1)	Most of the time (2)	Some of the time (3)	A little of the time (4)
32. During the past 4 weeks, how much of the time has your physical health or emotional problems interfered with your social activities (like visiting with friends, relatives, etc.)?				

How TRUE or FALSE is each of the following statements for you?	*Definitely true (1)*	*Mostly true (2)*	*Don't know (3)*	*Mostly false (4)*	*Definitely false (5)*
33. I seem to get sick a little easier than other people.					
34. I am as healthy as anybody I know.					
35. I expect my health to get worse.					
36. My health is excellent.					

A.3.2 Depression Anxiety Stress Scales - DASS21

Total number of questions: 21

Circle a number 0, 1, 2 or 3, which indicates how much the statement applied to you over the past week.	*0 (Did not apply to me at all)*	*1 (Applied to me to some degree, or some of the time)*	*2 (Applied to me to a considerable degree, or a good part of time)*	*3 (Applied to me very much, or most of the time)*
1. I found it hard to wind down				
2. I was aware of dryness of my mouth				
3. I couldn't seem to experience any positive feeling at all				
4. I experienced breathing difficulty (e.g., excessively rapid breathing, breathlessness in the absence of physical exertion)				
5. I found it difficult to work up the initiative to do things				
6. I tended to over-react to situations				
7. I experienced trembling (e.g., in the hands)				
8. I felt that I was using a lot of nervous energy				
9. I was worried about situations in which I might panic and make a fool of myself				
10. I felt that I had nothing to look forward to				
11. I found myself getting agitated				
12. I found it difficult to relax				

13. I felt down-hearted and blue				
14. I was intolerant of anything that kept me from getting on with what I was doing				
15. I felt I was close to panic				
16. I was unable to become enthusiastic about anything				
17. I felt I wasn't worth much as a person				
18. I felt that I was rather touchy				
19. I was aware of the action of my heart in the absence of physical exertion (e.g., sense of heart rate increase, heart missing a beat)				
20. I felt scared without any good reason				
21. I felt that life was meaningless				

A.4 Service Satisfaction Questionaries

A.4.1 Clinical Satisfaction (app and control group)

Total number of questions: 7

**Surgical Result**	Very dissatisfied	Dissatisfied	Neutral	Satisfied	Very satisfied
1. Overall, how satisfied are you with the results of your knee replacement surgery?					
2. How satisfied are you with the results of your knee replacement surgery for relieving your pain?					
3. How satisfied are you with the result of knee replacement surgery for improving your ability to perform regular activities?					
4. How satisfied are you with the results of the knee replacement surgery for improving your ability to do recreational activities?					
5. How satisfied are you with the results of the knee replacement surgery for meeting your expectations?					

**Recommendation**	Definitely yes	Possibly yes	Probably not	Certainly not	Not sure
6. Would you have this surgery again if it were required on another knee or joint?					
7. Would you recommend this surgery to someone else?					

A.4.2 Service Delivery Satisfaction (app and control group)

Total number of questions: 8

**Empowerment Experience**	Strongly disagree	Disagree	Neutral	Agree	Strongly agree
1. The service has improved my knowledge about my knee condition					
2. The service has improved my knowledge about the way I can better manage my knee condition					
3. The service has helped me stay engaged with my strengthening exercise (e.g. knee, upper body, lower body)					

**Interaction with Clinicians**	Very disappointed	Disappoin ted	Neutral	Satisfied	Very satisfied
4. How do you feel about the service provided by the physiotherapist in terms of the time given, contacting you, and helping you understand your conditions?					
5. How do you feel about the service provided by the surgeon in terms of the time given, contacting you, and helping you understand your conditions?					

**Observability**	Strongly disagree	Disagree	Neutral	Agree	Strongly agree
6. The effects of the service to manage my health condition is apparent to others					
7. I would recommend this service to other people					

**Overall Satisfaction**	Very disappointed	Disappointed	Neutral	Satisfied	Very satisfied
8. Overall how satisfied are you with the service?					

A.4.3 Technical Satisfaction: app, portal, wearable (app group only)

Total number of questions: 16

**Complexity**	Strongly disagree	Disagree	Neutral	Agree	Strongly agree
1. Overall I find the TKR app easy to use					
2. I sometimes find the TKR app frustrating to use					
3. Instructions on the TKR app are easy to understand and follow					
4. Using the TKR app is cumbersome					
5. I needed to learn a lot of things before I could get going with the TKR app					
6. I found the TKR app unnecessarily complex					
7. I think that I would need the support of a technical person to be able to use the TKR app					

8. I feel very confident using the TKR app					
9. I find the various functions in the TKR app well integrated					

**Compatibility**	Strongly disagree	Disagree	Neutral	Agree	Strongly agree
10. The TKR app is a tool that would be easy to incorporate into my daily routine					
11. The TKR app fits right into the way I like to manage my health					
12. Using the TKR app fits well with my lifestyle					

**Wearable**	Strongly disagree	Disagree	Neutral	Agree	Strongly agree
13. I found the Garmin activity tracker convenient and easy to use					
14. Information captured by the Garmin activity tracker was accurate and reliably reflected my activity					
15. I found the information presented by the Garmin activity tracker important					
16. The use of the Garmin activity tracker was conducive to my recovery					

A.5 Behavioural Measures

A.5.1 Self-Motivation Inventory (SMI)

Total number of questions: 40

	(a) extremely uncharacteristic of me	*(b) somewhat uncharacteristic of me*	*(c) neither characteristics nor uncharacteristic of me*	*(d) somewhat characteristic of me*	*(e) extremely characteristic of me*
1. I'm not very good at committing myself to do things.					
2. Whenever I get bored with projects I start, I drop them to do something else.					
3. I can persevere at stressful tasks, even when they are physically tiring or painful.					
4. If something gets to be too much of an effort to do, I'm likely to just forget it.					
5. I'm really concerned about developing and maintaining self-discipline.					
6. I'm good at keeping promises,					

	(a) extremely uncharacteristic of me	(b) somewhat uncharacteristic of me	(c) neither characteristics nor uncharacteristic of me	(d) somewhat characteristic of me	(e) extremely characteristic of me
especially the ones I make to myself.					
7. I don't work any harder than I have to.					
8. I seldom work to my full capacity.					
9. I'm just not the goal-setting type.					
10. When I take on a difficult job, I make a point of sticking with it until it's completed.					
11. I'm willing to work for things I want as long as it's not a big hassle for me.					
12. I have a lot of self-motivation.					
13. I'm good at making decisions and standing by them.					
14. I generally take the path of least resistance.					
15. I get discouraged easily.					
16. If I tell somebody I'll do something, you can depend on it being done.					
17. I don't like to overextend myself.					
18. I'm basically lazy.					
19. I have a very hard-driving, aggressive personality.					
20. I work harder than most of my friends.					
21. I can persist in spite of pain or discomfort.					
22. I like to set goals and work toward them.					
23. Sometimes I push myself harder than I should.					
24. I tend to be overly apathetic.					
25. I seldom, if ever, let myself down.					
26. I'm not very reliable.					
27. I like to take on jobs that challenge me.					
28. I change my mind about things quite easily.					
29. I have a lot of willpower.					
30. I'm not likely to put myself out if I don't have to.					
31. Things just don't matter much to me.					

	(a) extremely uncharacteristic of me	*(b) somewhat uncharacteristic of me*	*(c) neither characteristics nor uncharacteristic of me*	*(d) somewhat characteristic of me*	*(e) extremely characteristic of me*
32. I avoid stressful situations.					
33. I often work to the point of exhaustion.					
34. I don't impose much structure on my activities.					
35. I never force myself to do things I don't feel like doing.					
36. It takes a lot to get me going.					
37. Whenever I reach a goal, I set a higher one.					
38. I can persist in spite of failure.					
39. I have a strong desire to achieve.					
40. I don't have much self-discipline.					

A.5.2 Self-Efficacy Scale (SES)

Total number of questions: 5

I can manage to carry out my knee rehabilitation exercises, ...	*(1) very uncertain*	*(2) rather uncertain*	*(3) rather certain*	*(4) very certain*
1. ...even when I have worries and problems.				
2. ...even if I feel depressed.				
3. ...even when I feel tense.				
4. ...even when I am tired.				
5. ...even when I am busy.				

A.5.3 Self-Determination Questionnaire (SDQ)

Total number of questions: 4

Imagine that your main goal after your surgery is to be successful in your knee rehabilitation program. Below are four different reasons that you may feel this way. Please rate each of the statements for how well they represent the reason/s that you want to be successful in your knee rehabilitation program. There are no right or wrong reasons for this question, so please answer according to how you feel.

Please circle a number for each questionnaire item.

1. I want to be successful in my knee rehabilitation program…

Because somebody else wants me to, or because I’ll get something from somebody if I do – I probably wouldn’t want to do this if I didn’t get some kind of reward, praise or approval for it.

1	2	3	4	5	6	7	8	9
not at all because of this reason	→							Completely because of this reason

2. I want to be successful in my knee rehabilitation program…

Because I would feel ashamed, guilty or anxious if I didn't – I feel that I 'ought' to strive to do this.

1	2	3	4	5	6	7	8	9
not at all because of this reason	→							Completely because of this reason

3. I want to be successful in my knee rehabilitation program…

Because I really believe that it is an important goal to have – I endorse it freely and value it wholeheartedly.

1	2	3	4	5	6	7	8	9
not at all because of this reason	→							Completely because of this reason

4. I want to be successful in my knee rehabilitation program…

Because of the fun and enjoyment which this goal will provide me – the primary reason is simply my interest in the experience itself.

1	2	3	4	5	6	7	8	9
not at all because of this reason	→							Completely because of this reason

A.6 Health Economics

A.6.1 Health Care Provider Audit

What are the costs of running a group physiotherapy session?

1. Cost of staff (e.g. physiotherapist) per hour: $______
1. Cost of venue (e.g. room hire): $______
2. Administration costs: $______
3. Equipment costs: $______

What are the costs of running one-to-one physiotherapy session?

1. Cost of staff (e.g. physiotherapist) per hour: $______
2. Cost of venue (e.g. room hire): $______

3. Administration costs: $______
4. Equipment costs: $______

A.6.2 Patient Diary

Please answer the following questions to the best of your ability. Each question refers to the past 7 days. It is important that you complete this questionnaire once a week, on the same day.

Additional question for Week 1 only:

1. How many nights did you stay in hospital following your knee surgery? ____ nights
2. Select from the following that best reflect your carer's employment status:
   a. Employee, full time
   b. Employee, part time
   c. Self-employed
   d. Employee on sick leave
   e. Not in paid employment due to retirement
   f. Not in paid employment for other reasons
3. What is your carers occupation (e.g. bank manager, school teacher)? _____________

Question for Weeks 1 to 12:

1. In the past week, on how many days did you complete physiotherapy exercises at home?

   0 1 2 3 4 5 6 7

2. In the past week, on how many days were you absent from work? ____ days

   None, I currently do not work outside the home

3. In the past week, if you attended work, how well were you able to perform work activities?

   (unable to perform duties) (fully functioning)

   0 1 2 3 4 5 6 7 8 9 10

   I did not attend work in the past week

4. In the past week, on how many days was your carer absent from work due to your condition? ____ days

   None, my carer does not work outside the home

5. In the past week, if you needed help from friends or relatives with tasks that you usually carry out yourself, please note each episode of help received here.

**Who** provided help? (Friend, family, other)	**What help** did they provide? (e.g. cleaning, shopping)	**Number** of hours

6. In the past week, if you needed any professional services, with tasks that you usually carry out yourself, please note each episode of service received here.

Name of **Service** (e.g. cleaning, food/grocery delivery)	Number of **times** service needed

7. In the past week, if you needed someone to drive you somewhere please note each trip here:

Mode of **transport** (private car or taxi/Uber)	**Reason** for trip (e.g. clinic, GP, physiotherapy, visit friend)	**Number** of trips in past week

8. Please record any visits to your health care professional or any clinical services related to your knee replacement surgery that occurred in the past week.

**Date**	Brief **description** (e.g. GP visit, physiotherapy)	Number of **hours** of service	Tick (√) if this was a group session	Did your **carer** take you? (Yes/No)

9. Please record any non-clinical services received for your condition in the past week.

**Type** of service (e.g. gym, personal trainer, swimming pool, massage therapy etc.)	Number of **hours**	Was your **carer** with you (Yes/No)

10. Please record any medication purchases for your knee condition made in the past week.

**Type** of medication	Approximate **Quantity** purchased

11. Please record any other out-of-pocket expenses related to your condition incurred in the past week.

Out-of-pocket **expenses** (e.g. equipment, exercise tools)	Approximate **Cost** ($)

Addition question for Week 12:

12. When did you resume driving?

    Date _______ / I don't drive

Appendix B **Interview Questions**

Proposed questions for clinical trial

B.1 Questions for surgeons

1. How did you find the user interface?
2. What did you like/dislike about it? Why?
3. How frequently did you consult it?
4. Did you consult it or was it via your secretary or another person?
5. What information did you find most useful in terms of patient management?
6. Did you find the information provided clinically relevant?
7. Can you give me an example of how it might have helped in your patient management?
8. Do you believe that it helped some of your patients?
9. What aspects did patients like best/least?
10. What type of patients did it help the most?
11. Did you patients ever speak to you about their experience of using the app and or wearables?
12. What changes would you suggest?

B.2 Questions for physiotherapist

1. How easy/difficult did you find inputting exercise programs for your patients?
2. Where all the exercises you wanted in the program or are there other you would generally recommend?
3. Did you follow their progress and completion of exercises? Preoperative/recovery. How useful/interesting was that information to you.
4. If you had occasion to see the patients again in what did you use the information provided by the interface on exercise completion. Examples
5. Do you believe that the app helped patients complete their exercise program?
6. Were there challenges in helping people to access exercise programs in the app?
7. What changes would you suggest?
8. What types of patients did it work best for? (example)

B.3 Questions for carers

1. How easy did you find to use it?
2. Which features did you appreciate the most? Why?
3. Did you find you used it more preoperative than in the recovery? Why?
4. How did having access to an app help you in your role as carer?
5. Did it in anyway change the relationship between you and the patient? How?
6. Can you give me an example of how you would use it?
7. In what way did it help you as a carer?
8. Do you think it helped the patient?
9. Could they have used it without your assistance?
10. What changes would you suggest?

B.4 Questions for patients

1. What features aspects of the app did you like the best/least?
2. Which ones did you use the most?
3. Was it easy to use? Did you have any difficulty at all in using the app?
4. Did you find you used it more preoperative than in the recovery? Why?
5. In what way did the app help you at all in your preparation/recovery from surgery?
6. In what way did it help you? Example?
7. How did it help pre-op/post-op? In what way?
8. Did it change your relationship with your surgeon/physio in any way?
9. How did having an app make you feel? About your surgeon/hospital, about your preoperative/your recovery, exercises
10. Did you discuss the app or data from the app at all with your physio or surgeon?
11. Did you feel more empowered or in control of your preoperative/recovery? (example)
12. More motivated to do the exercises? (Example)
13. Did it in anyway change how you felt about your preparation /recovery (example)
14. Did it change in anyway what you did during your preparation/recovery (example)
15. What changes would you suggest?
